# Clinical features of 170 Chinese scabies cases and the therapeutic efficacy of sulfur ointment

**DOI:** 10.1371/journal.pntd.0014493

**Published:** 2026-07-08

**Authors:** Guiyuan Zhang, Mengxing Cui, Yue Zhang, Sijia Qin, Wenhui Du, Haibo Xie, Zhuo Zhu, Jing Xu, Bo Liang

**Affiliations:** 1 Department of Dermatology, The First Affiliated Hospital, Anhui Medical University, Hefei, Anhui, China; 2 Clinical Laboratory, The First Affiliated Hospital, Anhui Medical University, Hefei, Anhui, China; 3 Institute of Dermatology, The First Affiliated Hospital, Anhui Medical University, Hefei, Anhui, China; 4 Key Laboratory of Dermatology, Anhui Medical University, Ministry of Education, Hefei, Anhui, China; 5 Inflammation and Immune Mediated Diseases Laboratory of Anhui Province, Hefei, Anhui, China; 6 Anhui Provincial Institute of Translational Medicine, Hefei, Anhui, China; 7 Department of Parasitology, College of Veterinary Medicine, Sichuan Agricultural University, Chengdu, Sichuan, China; University of Notre Dame, UNITED STATES OF AMERICA

## Abstract

Scabies, caused by the *Sarcoptes scabiei* mite, is a highly contagious skin disease posing a global health challenge. This prospective study analyzed 170 scabies patients diagnosed at the dermatology outpatient clinic of the First Affiliated Hospital of Anhui Medical University between February 2024 and June 2025. We aimed to investigate the epidemiological characteristics, clinical manifestations, and therapeutic efficacy of sulfur ointment in scabies patients. Results showed that nodular lesions were the most common rash type (82.94%), with interdigital spaces being the most frequently affected site (91.18%). Misdiagnosis occurred in 69.41% of cases, with eczema being the most common misdiagnosis. Sulfur ointment treatment achieved complete cure in 54.71% of patients, partial improvement in 31.76%, and no response in 13.53%. The primary cause of treatment failure was non-compliance with medication protocols. Our study highlights the importance of comprehensive history-taking, accurate recognition of rash patterns, standardized treatment regimens, and simultaneous treatment of all exposed individuals to improve diagnostic accuracy and treatment outcomes for scabies.

## Author summary

Scabies is a highly contagious skin disease caused by *Sarcoptes scabiei*, which causes severe impact in subtropical and impoverished regions, particularly in settings such as dormitories, nursing homes, and prisons. The disease has traditionally been treated with permethrin, sulfur ointment, and benzyl benzoate. However, in recent years, resistance to permethrin has been reported. In China, sulfur ointment is widely used for scabies treatment due to its advantages of easy accessibility, low cost, and safety. This study analyzed the treatment outcomes and side effects of sulfur ointment in 170 patients diagnosed at the First Affiliated Hospital of Anhui Medical University between February 2024 and June 2025. The results showed that nodular lesions were the most common skin manifestation, and the interdigital spaces were the most frequently involved sites. After treatment with sulfur ointment, 54.71% of patients achieved complete cure, 31.76% showed partial improvement, and only 13.53% showed no improvement, which was mainly attributed to improper application of the medication.

## Introduction

Scabies, a highly contagious dermatological disease caused by the ectoparasite *Sarcoptes scabiei*, has emerged as a significant global public health concern. The disease exhibits particularly high prevalence rates in impoverished communities within tropical and subtropical regions, with reported infection rates reaching 32.1% to 74% in certain endemic areas [1]. Scabies has an especially pronounced impact on low-income families and young children [2]. Thornley *et al.* discovered that scabies prevalence was higher in early childhood care centers located in socioeconomically disadvantaged areas of Auckland [3]. The risk of scabies is markedly elevated in warm and overcrowded settings, such as nursing homes, dormitories, hospital wards, and correctional facilities, a single infection can rapidly escalate into an outbreak. Thus routine screening for scabies is strongly advised in high-density environments [4]. Scabies is divided into classic scabies and crusted scabies. In classic scabies, the number of adult female mites in the skin of patients typically remains below 20. However, patients with compromised mobility, poor hygiene, or immunodeficient states may develop to crusted scabies (Norwegian scabies) - a severe clinical variant characterized by hyperinfestation [5]. In such cases, the skin may harbor a very high mite burden, ranging from hundreds to millions of mites and eggs, leading to increased transmissibility [6,7].

## Materials and methods

### Ethics statement

This study was reviewed and approved by the Clinical Research Ethics Committee of the First Affiliated Hospital of Anhui Medical University, under approval number Anyi Yifuyuan Lunshen-PJ2025-01-43. All procedures performed in studies involving human participants were in accordance with the ethical standards of the institutional and/or national research committee and with the 1964 Helsinki declaration and its later amendments or comparable ethical standards. This study involves child participants, with all parents/guardians having signed written informed consent, formally consenting to their children’s involvement in the research.

### Research subjects

This study adopted a prospective single-center cohort design, recruiting 170 scabies patients from a tertiary hospital in Anhui Province, China. Comprehensive clinical data were gathered, and the characteristics of skin lesions and clinical symptoms were statistically analyzed. The epidemiological characteristics and possible pathogenic factors of scabies were explored, with a correlation made between misdiagnosis rates, misdiagnosed diseases, and the causes of misdiagnosis. The effectiveness of sulfur ointment was assessed, and the reasons for its failure in treatment were examined.

This study included 170 patients diagnosed with scabies at the Dermatology Department of the First Affiliated Hospital of Anhui Medical University between February 2024 and June 2025. The inclusion criteria were as follows: (1) Diagnosis based on established scabies criteria [8], supported by typical manifestations observed during full-body examination and confirmed through either history of confirmed exposure to scabies or laboratory identification of *S. scabiei* mites, eggs, or fecal pellets under microscopy; (2) Age 0–79 years, irrespective of gender; (3) Availability of complete clinical documentation. Exclusion criteria comprised: (1) Comorbid severe dermatological diseases or systemic diseases; (2) Incomplete follow-up data.

### Treatment protocol

All enrolled patients received standardized treatment with 10% sulfur ointment. The medication was applied once daily for three consecutive days, covering all skin surfaces below the neck with particular attention to affected areas. Patients were instructed to wash off the ointment after completing the treatment course. For patients with partial symptom relief but not complete resolution, a second course was administered after a 3-day interval. All personal items, including clothing and bed linens, were required to be washed in water at ≥60°C for 10 minutes.

### Efficacy assessment criteria

Therapeutic efficacy is categorized into three primary outcomes: cured (complete disappearance of skin rash with absence of pruritus), improved (no new lesions with significant alleviation in existing rash and pruritus, with or without residual scabies nodules), and ineffective (no improvement in rash or pruritus, post-treatment relapse, or treatment discontinuation due to sulfur ointment allergy [erythema, edema, or burning sensation]). Efficacy, adverse effects and relapse were assessed one month after the end of treatment.

## Results

### Clinical characteristics of the study subjects

The cohort comprised 132 males and 38 females (male-to-female ratio: 3.47:1), with a mean age of 26.9 ± 19 years. Analysis of diagnostic timing revealed that 24.12% (41/170) of patients were diagnosed within one month after symptom onset, while the majority (68.82%, 117/170) received diagnoses 1–6 months post-onset. Among the latter subgroup, nearly half (57/117) exhibited a symptom duration of approximately 1 month. Notably, 7.06% (12/170) of cases had a prolonged pre-diagnosis disease course of about one year. The mean interval from symptom onset to diagnosis for most patients was 2.47 ± 3.11 months. Microsoft excel 2016 and spss version 27 (IBM, Armonk, NY, USA) were used for data preparation and calculation. Chi-square tests were employed to assess the distribution sites and types of rashes. A p-value of <0.05 was considered statistically significant.

### Clinical characteristics of skin lesions

**Fig 1:**
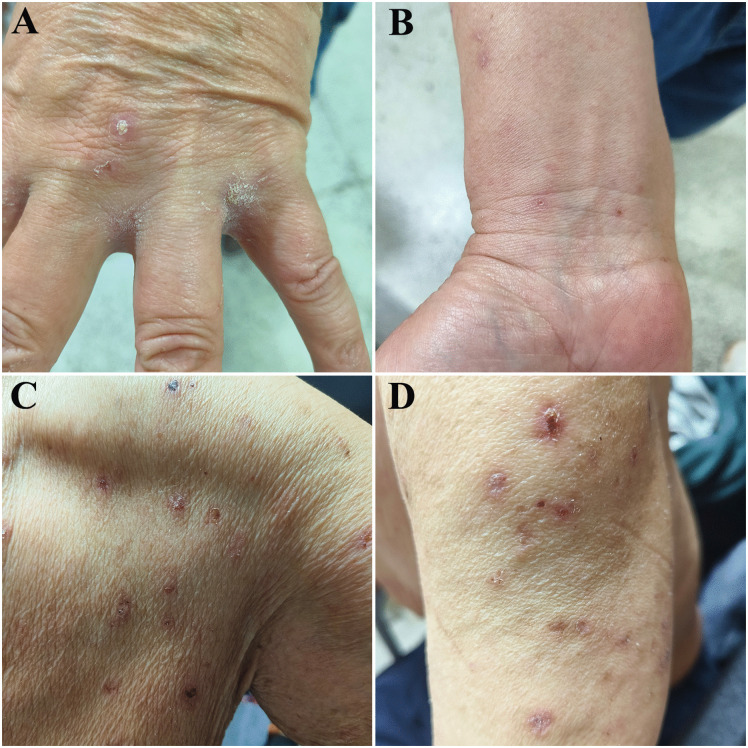
Clinical manifestations of scabies rash. (A) Rash in the interdigital spaces with scaling on the surface; (B) Rash on the flexor side of the wrist; (C) Rash on the left chest; (D) Rash on the upper arm.

**Fig 2:**
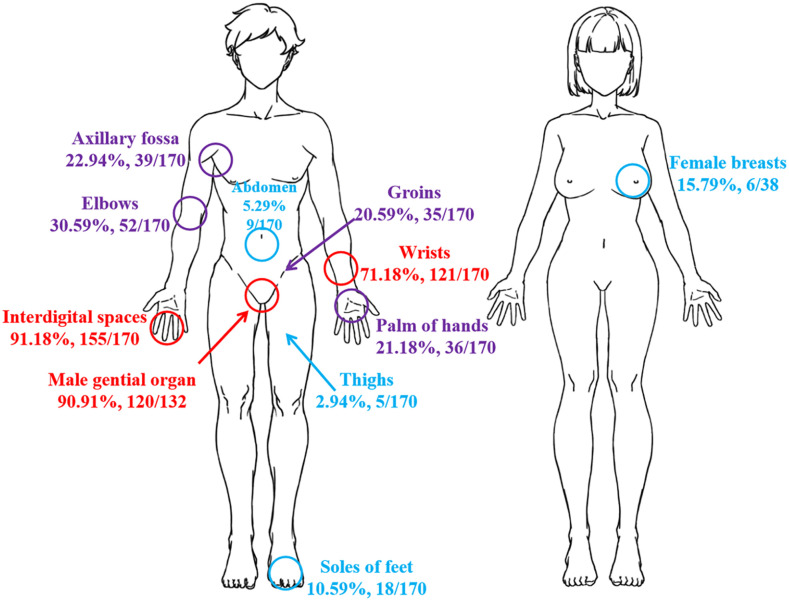
Schematic of the distribution of affected areas in 170 scabies patients. Color scale based on frequency: red = high, purple = moderate, blue = low.

In this study, over 90% of patients had rashes in the interdigital spaces or male genital area. Rashes were also commonly observed on the wrists (71.18%, 21/170), elbows, axillary fossa, palms, and groins, with incidence rates ranging from 20% to 30% (Fig 1). Some patients also had rashes on the soles of their feet, abdomen, thighs, or female breasts. A total of 15.29% (26/170) of patients experienced generalized rashes. The frequency and proportions of rashes in different areas were shown in Fig 2. The distribution of rashes across body sites showed a highly significant difference (χ² test, p < 0.001), with the highest incidence rates in the interdigital spaces (91.18%) and male genitalia (90.91%). Pairwise comparisons with Bonferroni correction revealed no significant difference between these two regions (p = 1.000), but both had significantly higher incidence rates than other areas (all p < 0.001).

Regarding rash characteristics, 82.94% (141/170) of patients had nodules, with one-third of male patients exhibiting scrotal nodules. Papules and vesicles were also commonly observed in 60%-80% of patients. A chi-square test comparing the incidence of papules (78.24%), vesicles (68.24%), and nodules (82.94%) revealed a statistically significant difference (p < 0.05). Further pairwise comparisons indicated a significant difference between vesicles and nodules (p = 0.0069), but no significant difference between papules and vesicles (p = 0.1491) or between papules and nodules (p = 1.0). Notably, 3 patients developed pustules. Almost all patients experienced itching, except for one with a spinal injury, who did not, likely due to sensory impairment or a weakened immune response. These findings confirm the polymorphic nature of scabies lesions as a key clinical feature.

### Epidemiological analysis of infection sources

Around one-third of patients acquire scabies through transmission from family members, highlighting the need for joint treatment of family members to break the transmission chain. The other one-third of patients are infected in communal settings, such as dormitories, hospitals, nursing homes, and so on. When scabies cases are identified in these places, it is crucial to strengthen environmental disinfection, conduct personnel screening, and, if necessary, carry out mass medication to prevent the spread of the outbreak. The infection source for the remaining one-third of patients remains uncertain. Moreover, a small number of patients contracted scabies in places like hotels, hot springs, gyms, amusement parks, or rental apartments.

### Laboratory confirmation of scabies diagnosis

**Fig 3:**
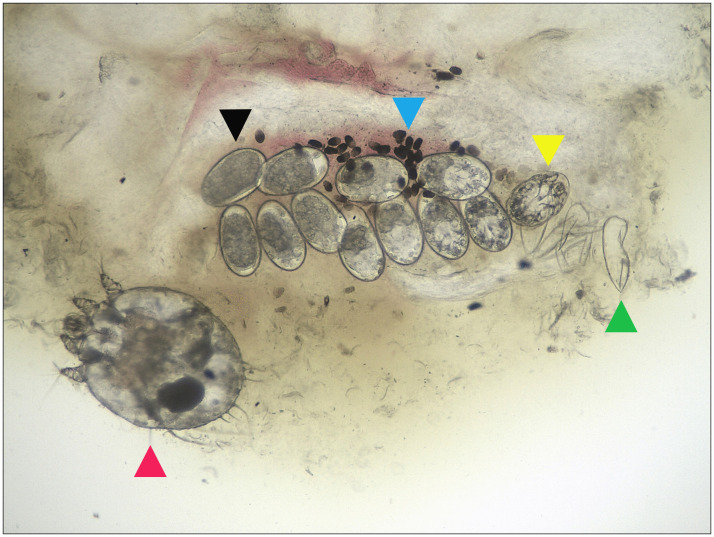
Microscopic identification of *S. scabiei.* Microscopic image of a scabies patient’s thigh inner side rash burrow, with the adult scabies mite (red arrow), fertilized eggs (black arrow), fecal particles (blue arrow), mature eggs (yellow arrow), and egg shells (green arrow) (400 × magnification).

Conventional microscopic examination of skin scrapings was performed in all 170 clinically diagnosed scabies cases. As demonstrated in Fig 3, the diagnostic evaluation revealed characteristic mites, eggs, or burrows observed under the microscope. Scabies mite eggs have thin, semi-transparent shells, and feces appear as dark brown or black particles. The laboratory testing yielded positive results in 97 cases, corresponding to a positive rate of 57.06% (97/170).

### Misdiagnosis conditions

Scabies was misdiagnosed in 69.4% (118/170) of patients, with eczema being the most common misdiagnosis. Many of these patients experienced single or repeated misdiagnoses, which may be related to population heterogeneity. This study found that approximately one-third of patients were misdiagnosed as eczema, followed by around one-quarter being misdiagnosed as allergic dermatitis or urticaria. Furthermore, a small proportion of patients were misdiagnosed with conditions such as folliculitis, papular urticaria, prurigo, flat warts, or fungal infections. The specific details of misdiagnosis can be found in Table 1.

**Table 1:** Misdiagnosed Conditions in Scabies Patients.

Misdiagnosis	N = 170 n(%)
Eczema	52(30.59)
Allergic dermatitis	30(17.65)
Urticaria	11(6.47)
Folliculitis	5(2.94)
Papular urticaria	4(2.35)
Prurigo	3(1.76)
Flat warts	2(1.18)
Fungal infections	1(0.59)

Note: n (%) number and percentage of patients.

### Treatment efficacy evaluation of sulfur ointment therapy

**Fig 4:**
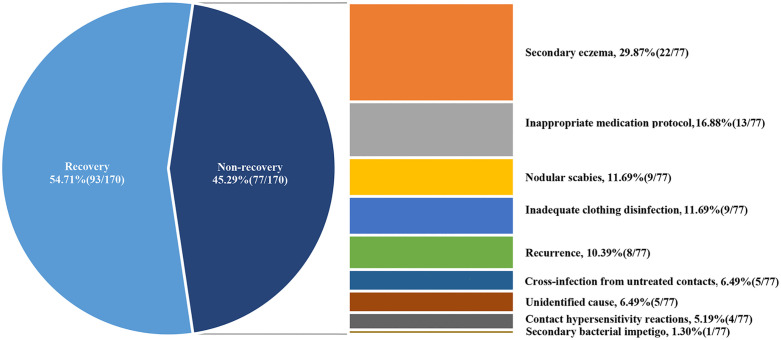
Therapeutic outcomes of sulfur ointment treatment in 170 scabies patients. The cure rate for scabies treatment with sulfur ointment is 54.71%, while the non-cure rate is 49.29%. The reasons for non-cure are as follows: secondary eczema, inappropriate medication protocol, nodular scabies, inadequate clothing disinfection, recurrence, untreated close contacts, contact hypersensitivity reactions, unknown causes, and secondary bacterial impetigo.

An analysis of 170 scabies patients treated with sulfur ointment revealed that over half of the patients were cured, approximately one-third showed significant improvement, and the total effective rate was 86.47%. The main reasons for treatment improvement without full recovery were eczema reactions or persistent scabies nodules. In this study, 13.53% of patients experienced treatment failure, with the failure rate among elderly patients being 25%, significantly higher than the general population. Common reasons for treatment failure include non-compliance with medication, such as not reapplying the ointment after bathing or applying it only locally. Among the overall treatment failure cases, there were 8 relapses, 4 cases of contact hypersensitivity, and 1 case of secondary bacterial impetigo caused by scratching, which later resolved with treatment using compound ketoconazole cream and chloramphenicol eye ointment. The specific causes for the failure to recover are shown in Fig 4. Approximately 25% of patients experienced eczema, dry skin, or allergic reactions following sulfur ointment treatment, with symptoms improving after stopping the medication and providing symptomatic care.

## Discussion

### Clinical characteristics of skin lesions

This study analyzed the clinical characteristics of 170 scabies patients, focusing on the rash distribution and morphological features. Studies have shown that scabies mites tend to infest areas with higher temperatures and thinner stratum corneum, including the interdigital spaces, scrotum, wrists, elbows, axillary fossa, and groin, match this characteristic. This finding indicates that clinical diagnosis and treatment should focus on examining these areas to enhance the early detection rate of scabies. Previous studies have hypothesized that scabies mite predilection sites may relate to skin lipid composition [9]; however, our data did not support a lipid-based distribution pattern. Moreover, 10% to 20% of patients exhibit rashes on the soles or palms. This finding extends the recognition of atypical distribution described in the literature [10], indicating that adult patients may also develop palmoplantar lesions similar to those seen in infants. Clinicians should be aware of this atypical distribution to avoid misdiagnosis. Severe itching is the primary symptom of scabies. This study found that nearly all patients experienced itching, except for one patient with a spinal injury. It is particularly important to note that all 13 elderly patients (aged 62–79 years) included in this study experienced pruritus, differs from the previous observation and may reflect the clinical population’s heterogeneity [11]. The exact mechanism of itching caused by scabies remains unclear, but it may be linked to the increase in non-histaminergic itch mediators in the skin after scabies mite infection [12]. Scratching damages the skin barrier, facilitating the occurrence of secondary bacterial infections. Acute rheumatic fever, rheumatic heart disease, post-streptococcal glomerulonephritis, and invasive bacterial infections are critical complications caused by pyogenic streptococcus, leading to substantial public health burdens and potentially severe health outcomes [6,13]. Therefore, early diagnosis and effective treatment of scabies not only help relieve symptoms but also prevent severe complications and reduce societal health risks. This study enhances the understanding of the clinical features of scabies, especially in terms of rash distribution, symptoms in elderly patients, and potential complications, offering valuable insights for clinical treatment.

### Laboratory confirmation of scabies diagnosis

The most advanced method for laboratory diagnosis of scabies involves examining lesion samples under a light microscope to identify scabies mites, eggs, or fecal particles [14]. The positive detection rate in 170 patients was 57.06%, which is consistent with previously reported sensitivity [15], confirming the feasibility of skin scraping in clinical practice. However, the adult mites are few in number and sparsely distributed [16], making direct detection challenging. This study found that the detection rate of eggs was higher than that of adult mites, indicating that in microscopic diagnosis, attention should be directed towards mite fragments, eggs, and fecal particles. A large number of characteristic egg shells or fecal particles, coupled with clinical symptoms like pruritus and rash, should prompt consideration of scabies even in the absence of adult mites. This study offers a detailed description of the microscopic features of scabies mite eggs and feces, aiding in the enhancement of detection rates under the microscope and providing evidence for clinical diagnosis.

### Misdiagnosis conditions

This study revealed that scabies is frequently misdiagnosed, with the majority of patients having experienced misdiagnosis during their medical visits, and some even being misdiagnosed multiple times, resulting in a disease course lasting more than a year and severely impacting their quality of life. The causes of scabies misdiagnosis are varied and multifactorial.

**Fig 5:**
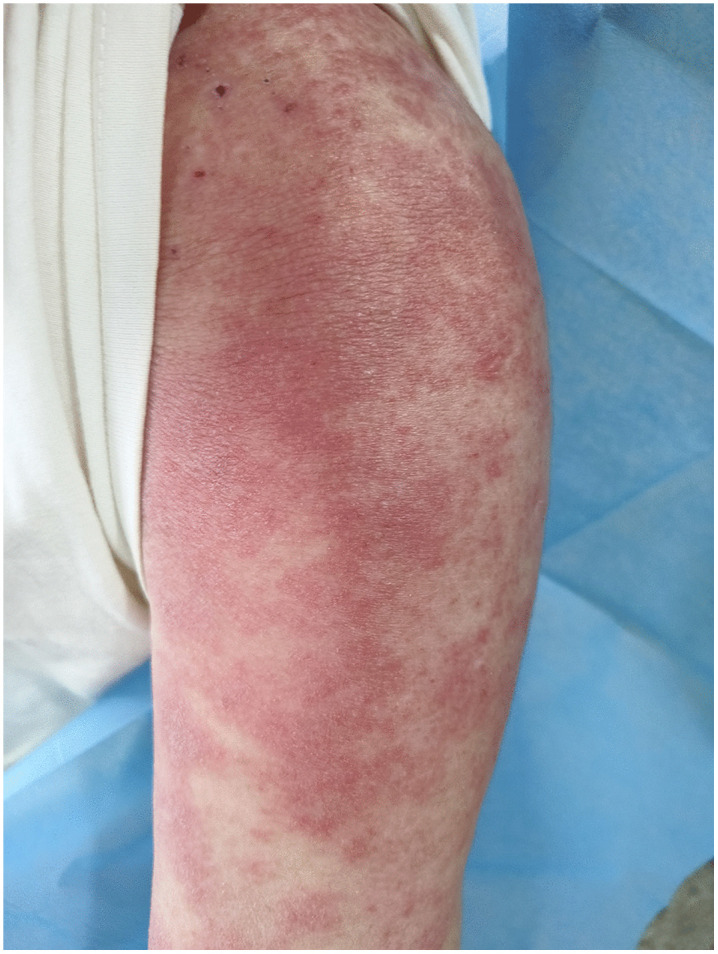
A scabies patient with secondary eczema-like changes due to scratching and excessive washing.

Scratching and over-washing can result in secondary eczematous-like changes, which are easily misdiagnosed as eczema, allergic dermatitis, or other diseases, thus delaying treatment (Fig 5). Furthermore, a negative microscopic examination result can complicate the diagnosis, and the atypical locations and types of rashes often lead to misdiagnosis. For example, severe crusted scabies is prone to being misdiagnosed as eczema [17]. Special populations face additional diagnostic challenges. In infants, rashes tend to occur on the face, scalp, palms, and soles [18], where they may present as papules, nodules, vesicles, or pustules. Scabies in elderly or immunocompromised patients can have varying presentations, often mimicking other pruritic skin diseases, leading to prolonged symptoms and increased severity [19]. Furthermore, abuse of topical corticosteroids may cause immune suppression, promoting scabies mite proliferation while altering the morphology of the rashes, further leading to a misdiagnosis [12,20]. Additionally, scabies mites and symbiotic bacteria can jointly secrete complement-inhibiting proteins, escaping immune clearance by the host [21], causing secondary bacterial infections that alter the lesion morphology and delay the onset of typical clinical symptoms, leading to misdiagnosis [12].

This study indicates that scabies is often misdiagnosed, with the majority of patients having experienced misdiagnosis— some even multiple times—leading to a disease course lasting more than a year and significantly affecting their quality of life. From a clinical management perspective, this suggests that doctors should improve their sensitivity to atypical manifestations, promptly identify suspected cases, and provide effective treatment to shorten the disease course and enhance patients’ quality of life. From a public health standpoint, early diagnosis and treatment can help reduce scabies transmission, mitigate the risk of community outbreaks, prevent secondary infections, and ease the overall societal health burden.

### Causes of sulfur ointment treatment failure

This study found that the effectiveness rate of sulfur ointment treatment was 86.47%, and 13.53% of patients showed no response after use. Inappropriate medication use was the most common reason for treatment failure. Some patients failed treatment due to not reapplying the medication after bathing, using insufficient amounts, or applying it only to local lesions (not whole-body application). The recommended dose for adults is at least 30g for a single full-body application [22]. Additionally, nine patients had treatment failure or recurrence due to inadequate clothing disinfection, highlighting the need for proper disinfection of the patients’ clothing [22–24]. Among patients with treatment failure, 5 cases were attributed to the failure to treat close contacts (especially those living together long-term) simultaneously. All family members and close contacts, even without symptoms, should undergo simultaneous prophylactic treatment to prevent them from becoming potential sources of reinfection [11]. A controlled study by Mumcuoglu *et al*. demonstrated that the cure rate in the household simultaneous treatment group was almost twice that of the individual treatment group, further validating the necessity of group interventions [25].

Twenty-three patients developed eczema following scabies treatment, likely related to the biological traits of scabies mites, skin immune responses, and scratching behaviors. Scabies mite activities, along with their fecal particles and secretions, persistently irritate the skin, triggering inflammatory responses. Meanwhile, severe itching drives patients to scratch persistently, damaging the skin barrier and facilitating the invasion of external irritants (e.g., bacteria, allergens), which exacerbates eczema. The study observed that for patients who showed improvement but did not fully recover after the initial treatment, a second dose significantly alleviated symptoms or resulted in full recovery. Additionally, overuse of sulfur ointment and similar medications can trigger contact dermatitis, manifesting as eczema-like reactions.

In China, sulfur ointment was the traditional first-line treatment for scabies, followed by permethrin or lindane cream, respectively. Sulfur ointment was affordable, easily accessible, and effective in inhibiting the growth of the scabies mites [26]. Despite its strong odor and greasy texture, it was widely utilized in primary healthcare, particularly for infants and pregnant women due to its safety. Permethrin was more expensive and not easily available in some areas, limiting its widespread use within China. Lindane cream was potent against scabies but could cause neurotoxicity (such as seizures) due to potential transdermal absorption [27]. It was not the first choice and was reserved for severe or drug-resistant cases in adults who could follow strict medical guidelines.

Therefore, based on our findings, we suggest that sulfur ointment can serve as a first-line drug for treating scabies. Previous studies have reported that although permethrin is a first-line treatment, its cure rate is only 27%, and its poor efficacy has been attributed to resistance [28]. Benzyl benzoate has a cure rate of 87%, which is similar to sulfur ointment. Previous reports have also indicated that young patients respond better to benzyl benzoate, crotamiton, and permethrin than elderly patients [26,29]. Our study showed that elderly patients responded less effectively to sulfur ointment compared with younger patients. In conclusion, age might be a key factor influencing scabies treatment outcomes, rather than the specific drug used. Apart from using sulfur ointment for scabies treatment, Lake et al. discovered that mass drug administration (MDA) is very effective in lowering the prevalence of scabies and impetigo [30]. Engelman *et al.* suggested that pilot MDA control measures should be implemented in areas where community scabies prevalence is at or above 10% [31]. The proposed protocol involves taking 2 doses of ivermectin (200μg/kg) orally, with a 7–14-day interval, and conducting 3–5 rounds of MDA annually until the community prevalence decreases to less than 2%.

This study, with a small sample size (n = 170) and a single treatment intervention (sulfur ointment), may have limited statistical power and external validity. Future research should include multicenter, large-sample randomized controlled trials to address potential biases and compare the efficacy of sulfur ointment against other first-line anti-scabies treatments. The study found that scabies rashes are often seen in areas with thinner epidermis and higher temperatures, such as the webbing of fingers and the male genitalia, while atypical rashes should also be suspected. All patients had accompanying pruritus symptoms. During microscopic examination, in addition to detecting scabies mites, attention should be paid to scabies eggs and feces to enhance detection rates. Scabies symptoms are easily confused with those of other diseases, and many patients are misdiagnosed prior to the correct diagnosis. Sulfur ointment shows significant effectiveness in treating scabies, with occasional skin irritation but no other adverse reactions, thus it can be used as a first-line therapeutic agent.

## Supporting information

S1 Table Demographic and clinical characteristics, prior treatments, and misdiagnosis status of 170 Chinese scabies patients. (XLSX)
